# Recent Progress in Pyro-Phototronic Effect-Based Photodetectors: A Path Toward Next-Generation Optoelectronics

**DOI:** 10.3390/ma18050976

**Published:** 2025-02-21

**Authors:** Vishwa Bhatt, Min-Jae Choi

**Affiliations:** Department of Chemical and Biochemical Engineering, Dongguk University, Pildong-ro 1-gil, Jung-gu, Seoul 04620, Republic of Korea; vbhatt23@dgu.ac.kr

**Keywords:** pyroelectricity, pyro-phototronic effect, self-powered photodetection, broadband photodetection

## Abstract

Since photodetectors are widely used in a variety of applications, such as imaging, optical communication, security and safety, motion detection, environmental sensing, and more, they are a crucial part of many technologies. The performance of photodetectors has significantly improved due to the advanced development of third-generation semiconducting materials caused by the novel pyro-phototronic effect. This effect; induced by localized heating under pulsed incident light, enhances the generation, separation, and collection of charge carriers within photodetectors. The combined pyroelectric and photoelectric effects resulting from this process are collectively termed the pyro-phototronic effect. It is crucial to understand how the pyro-phototronic effect affects the optoelectronic processes that take place during photodetection. This review addresses the latest advancements in photodetector performance by presenting the pyro-phototronic effect for a range of semiconductors. We provide a comprehensive summary of the pyro-phototronic effect in different semiconducting materials and outline recent developments in photodetectors.

## 1. Introduction

Photodetectors (PDs) are not only concerned about converting light into an electrical signal but also about the amplitude, sensitivity, spectral responsivity, and response–recovery time [1]. PDs have been employed in the industry and research laboratories to measure light intensity since 1900 [2]. Photoconductivity was first discovered by Willoughby Smith in 1873. Later, in 1940, Russell Ohl at Bell Labs discovered PDs based on the silicon p-n junction [3,4]. The diverse range of PDs has been a key component in artificial intelligence (AI) and Internet of Things (IoT) technologies owing to their extensive applications including light communication, automotive engineering, military, image sensing, thermal imaging, night vision, biochemical analysis, motion detection, space exploration, and many more [5,6,7,8,9,10]. PDs are one of the most widely used devices in various technologies, spanning the electromagnetic spectrum from very short X-rays to radio waves that are thousands of meters long. PDs can be classified in various ways, with the most common method being based on their ability to detect either a specific wavelength or a broad wavelength range. Various semiconducting materials include metal oxides, metal sulfides, metal dichalcogenides, compound semiconductors, perovskites, hybrid materials, graphene, black phosphorus, nitrides, chalcogenides, halides, and many more. Apart from the conventional PDs, colloidal quantum dots, one- (1D), two- (2D), and three-dimensional (3D) semiconducting materials have demonstrated significant advancements in enhancing PD performance. These semiconducting materials pose dimension-dependent advantageous characteristics. The enhancement of photodetection performance parameters is sensitivity, detectivity, and photoresponsivity. The key performance parameters are driven by excellent light absorption capability, charge carrier generation, and collection efficiency. The balanced integration of these three parameters enables PDs to exhibit superior photodetection characteristics. The absorption losses, recombination losses, and poor charge collection are the primary factors contributing to suboptimal photodetection performance. Various effect-based improvements have been observed for a wide range of semiconducting materials including the photovoltaic, photoconductive, photogate, and surface plasmonic effects [11,12,13,14,15,16].

The pyroelectric effect is a lesser-known property of certain anisotropic semiconducting materials. This effect can be characterized by the temperature dependence of spontaneous polarization. The pyroelectric effect has been utilized in various applications including energy harvesting, hot image detectors, intruder alarms, and temperature sensors [17,18,19,20]. The pyroelectric effect in light-absorbing semiconducting materials was observed by Wang et al. in 2015, introducing a new mechanism for enhancing ultraviolet (UV) photodetection through the light-induced pyroelectric effect in ZnO nanowires (NWs). The pyroelectric potentials are induced by temperature changes (ΔT) across the semiconducting material owing to non-central symmetric crystal structures of wurtzite ZnO NWs [21,22]. This novel approach altered the charge-transport processes under light absorption in ZnO by facilitating pyroelectric polarization [23]. Since then, various UV light PDs based on ZnO have been extensively developed utilizing light-induced pyroelectric effects known as the pyro-phototronic effect (PPE) [24,25,26,27,28]. These reports analyzed the role of PPE in enhancing photodetection performance by generating additional charge carriers under localized heating caused by light absorption. The built-in electric field can be modulated by combining pyroelectric and photoelectric effects. These potentials are generated in response to changes in the external environment, which enhances the existing photogenerated potential. This increase in the internal electric field accelerates the separation and transport of charge carriers, thereby improving PDs’ performance. Among these mechanisms, the pyroelectric effect facilitates the regulation of the material’s surface temperature in response to variations in illumination [29,30]. This effect induces the generation of thermoelectric potential without needing external fields or pressures on device materials. This feature offers greater convenience for practical applications. Furthermore, PPE can generate pyroelectric potential under illumination that exceeds the material’s bandgap. It enables a significant broadening of the PD’s optical response range and enables efficient wide-spectrum detection [31,32]. For instance, Bhatt et al. leveraged the PPE of Ag_2_S colloidal quantum dots (CQDs) which significantly enhanced the photocurrent. It increased the detection capability of the CQD/silicon PDs by 17-fold, achieving 4.1 × 10^10^ Jones at 980 nm compared to 2.3 × 10^9^ Jones for devices lacking the PPE [33]. Luo et al. utilized the PPE of (S-BPEA)_2_FAPb_2_I_7_ (S-BPEA = (S)-1-4-Bromophenylethylammonium, FA = Formamidinium). It endows chiral perovskite with remarkable broadband circularly polarized luminescence (CPL) detection performance within the range of 405–785 nm [34]. The researchers focused on enhancing photodetection performance by considering the synergistic coupling of pyroelectric and photoelectric effects.

The review article introduces the pyro-phototronic effect (PPE) to enhance photodetection. PPE is a promising phenomenon that can enhance photodetection performance by generating additional charge carriers across the junction. PPE enhances charge carrier processes by utilizing localized heating from pulsed light and combining pyroelectric and photoelectric qualities. This technique improves sensitivity, detectivity, and reaction time by using light-induced pyroelectric polarization in semiconducting materials to produce extra charge carriers, in contrast to traditional photodetection techniques. By providing a novel technique to enhance light absorption and charge transport efficiency across a broad spectrum, the method offers a breakthrough in next-generation photodetectors and eventual advances in imaging, optical communication, and environmental sensing technologies. These additional charge carriers improve charge carrier generation efficiency, potentially enhancing their functionality and making them highly efficient for developing next-generation PDs. This review article analyzes the various development phases, classifications, and principal performance indicators of photodetectors (PDs) that utilize the pyro-phototronic effect (PPE). The detail explains how optical detection is elevated by combining a pyroelectric and photoelectric effect that improves charge carrier processes. This review summarizes the progress on materials such as nanowires, quantum dots, perovskites, and chiral materials for broad- and narrowband photodetection. It also addresses some of the PPE-assisted charge collection approaches that broaden the optical response range. The experiments described in this review article are proof of these technologies. We detail the weaknesses associated with PPE photodetectors, recommend areas for further examination, and investigate the possibilities of utilizing PPE photodetectors in conjunction with artificial intelligence and the Internet of Things for more sophisticated sensing technologies. Lastly, this review stresses the importance of developing theoretical models that would optimize the physical mechanisms of PPE for efficient devices to be used in communications, security, space, and biomedicine.

In this comprehensive review, we discussed the latest advancements in PPE-based PDs in terms of performance parameters. It also provides an in-depth analysis of PPE-driven charge generation, transport, and collection mechanisms. Additionally, we examine PPE induced in new semiconducting materials demonstrating photodetection enhancement across a broad range of light. In contrast to conventional material-based or spectrum responsivity classifications, the paper presents a new classification methodology for photodetectors (PDs) based on the pyro-phototronic effect (PPE), which is driven by functionality. It emphasizes PPE-driven improvements and classifies PDs according to their charge carrier dynamics and energy harvesting capabilities. This new classification improves PD performance parameters including sensitivity, detectivity, and spectrum response by considering the complementary impacts of pyroelectricity and photoelectricity. By proposing a systematic approach that combines experimental validation, theoretical modeling, and AI-assisted simulations, this work provides a roadmap for optimizing PD efficiency, scalability, and integration into next-generation smart sensing and quantum optoelectronic applications. The review paper is organized as follows: First, it briefly discusses the fundamentals of PPE-based PDs, including their working mechanisms and performance parameters. Next, it delves into the properties of PPE in different junctions. Following this, the paper examines PPE in both broadband and narrowband PDs. Finally, it thoroughly explores the challenges faced by current PPE-induced PDs and considers future prospects in various aspects.

## 2. Fundamentals of the Pyro-Phototronic Effect-Based Photodetectors

**Figure 1:**
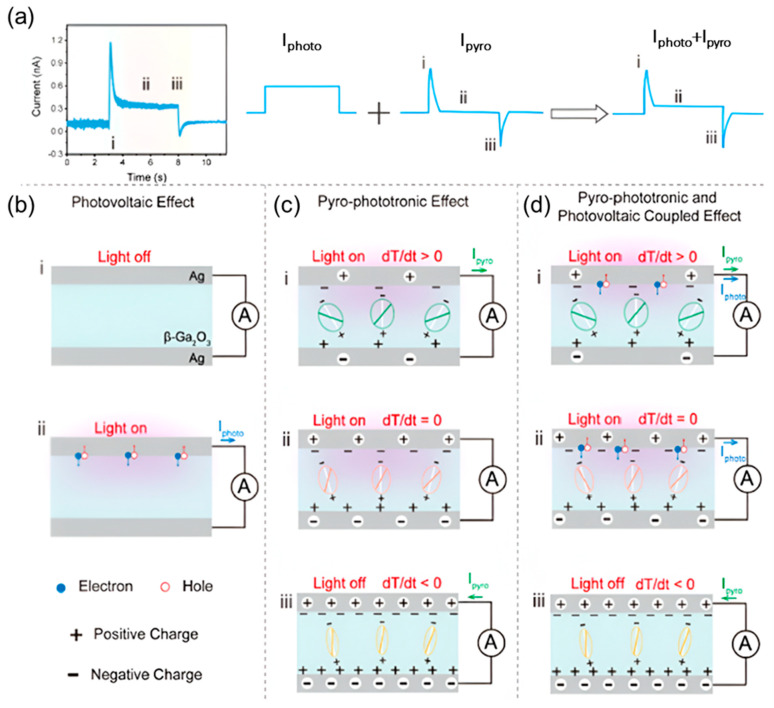
Mechanism of the PPE in the Ag/β-Ga_2_O_3_ PD. (**a**) I–t response of Ag/β-Ga_2_O_3_ PD and demonstration of the transient photocurrent induced by the PPE and photovoltaic effect. Schematic showing a working mechanism of the (**b**) photovoltaic effect, (**c**) PPE, and (**d**) coupling of the pyro-phototronic and photovoltaic effects in the Ag/β-Ga_2_O_3_ PD [39].

Figure 1a illustrates the detailed schematic of the behavior of PDs under the PPE. Figure 1b–d explains the fundamental working mechanisms of PDs under the photovoltaic effect, PPE, and the coupled pyro-phototronic–photovoltaic effect. The I_photo_ is generated by the photovoltaic effect where photocurrent is mainly due to space charge region formation across the Ag and Ga_2_O_3_ Schottky junction (Figure 1b). Figure 1c describes the pyroelectric effect during which light illumination induces oscillation of electric dipoles due to an increase in light-induced ΔT [18,35]. When light exposure is removed, self-polarization occurs, and free carriers flow through the conductive mechanism. It generates a sharply pulsed pyro-phototronic current. When light is applied to PDs under coupled pyro-phototronic and photovoltaic effects, PDs experience a slower increase in the photovoltaic effect combined with a faster PPE, ultimately producing a sharp current [36,37,38]. Increased localized temperature (dT/dt > 0) in crystal promotes the oscillatory state of internal electric dipoles. These electric dipoles reduce internal polarization by decreasing electrostatic induction charges. Thus, when the directions of pyro-phototronic and photovoltaic currents are identical, a high magnitude of current is generated. Once the device stabilizes under light exposure, the PDs reach the thermal equilibrium (dT/dt = 0), i.e., the PPE diminishes, and the photovoltaic effect is dominant. Furthermore, when the light is turned off, a rapid drop in localized temperature (dT/dt < 0) occurs. And suddenly increased polarization enhances electrostatic induction charges generating current in the opposite direction. Once the PDs reach an equilibrium state, the PDs exhibit a stable dark current.

### 2.1. Performance Parameters of Pyro-Phototronic Effect-Induced Photodetectors

Some basic PD performance parameters need to be analyzed to understand the merit of the PDs. The PD performance parameters include sensitivity, detectivity, spectral responsivity, LDR, EQE, and rise/fall time. The performance parameters are discussed in detail below [40]:

Sensitivity (S): The sensitivity of the PDs indicates the relative change in the photocurrent (pyro–photo) and dark current. For highly efficient photodetection, a lower dark and higher light current are expected. The sensitivity of the photoelectric effect and PPE are defined as follows:

(1) S(photo)=I(photo)−IdarkIdark

(2) S(photo+pyro)=I(photo+pyro)−IdarkIdark

where I_photo_, I_photo+pyro_, and I_Dark_ are the photo-, pyro-phototronic, and dark currents.

Responsivity (R_λ_): Responsivity is one of the most important parameters for evaluating PD performance. Responsivity represents the magnitude of the photocurrent (pyrocurrent + photocurrent) under light exposure of a certain power intensity. A higher photocurrent (pyrocurrent + photocurrent) even at a low power intensity indicates good responsivity. The formula for the responsivity is mentioned below:

(3) Rphoto=I(photo)P

(4) Rphoto+pyro=I(photo+pyro)P

where P presents the light intensity power.

Detectivity (D*): Like responsivity, detectivity measures the sensitivity of capturing a weak signal. Detectivity relies on responsivity, noise current, and power intensity. Hence, the capability of detecting a weak signal is defined as detectivity. The formula for detectivity is given below:

(5) $$D_{\left(photo\right)}^{*}=\left(\frac{R_{\left(photo\right)}\sqrt{A}}{I_{N}}\right)$$

(6) $$D_{\left(photo+pyro\right)}^{*}=\left(\frac{R_{\left(photo+pyro\right)}\sqrt{A}}{I_{N}}\right)$$

where I_N_ represents the noise current. The noise current is given by $\left(I_{N}\right)=V_{N}\times S$ where V_N_ and S are the voltage noise (in V/Hz^−^^1/2^) and sensitivity of the transimpedance amplifier, respectively.

Linear dynamic range (LDR): LDR is also one of the most important parameters that define the linearity of PDs. The magnitude of the LDR depends mainly on the photocurrent (pyro+photo) and dark current. The formula for LDR is mentioned below:

(7) LDRphoto=20LogIphotoIdark

(8) LDRphoto+pyro=20LogIphoto+pyroIdark

External quantum efficiency (EQE): The EQE is defined as the number of electrons–holes collected per photon incident. EQE can be calculated based on the responsivity using the formula mentioned below:

(9) $${EQE}_{photo}=\left(\frac{hc}{q\lambda }\right)R_{\left(photo\right)}$$

(10) $${EQE}_{photo+pyro}=\left(\frac{hc}{q\lambda }\right)R_{\left(photo+pyro\right)}$$

where q indicates the charge, λ indicates the wavelength of light, and h and c denote Planck’s constant and the speed of light.

Rise/fall time (t_r_/t_f_): In addition, PD performance parameters mainly depend on the dark current, photocurrent, and power intensity. But it is also most important to analyze how much time the PDs require for the transition from the off to the on state and vice versa. The rise/fall time depends mainly on the generation and recombination rate of the electron–hole pair. Depending on the junction properties, the rise/fall time of the PDs can be determined. The rise/fall time is also affected by the equivalent intrinsic RC constant and defect states existing in the semiconducting material. The rise time is calculated as the time required for the rising edge to reach 10 to 90% of the response. The fall time is calculated based on the time required for the falling edge to reach 90 to 10% of the response. The rise/fall time depends on the individual characteristics of the PDs. If the rising/falling edge does not exhibit a sharp transition, it is difficult to calculate the rise/fall time using the conventional formula. In such cases, the single-/bi-exponential function must be applied to calculate the valid rise/fall time. To evaluate the existing time constants, the transient responses must be fitted using the bi-exponential function as mentioned below [41]:

(11) $$I{\left(t\right)}_{Rise}=I_{0}+A_{1}\left(1-e^{-t/\tau_{r1}}\right)+A_{2}(1-e^{-t/\tau_{r2}})$$

(12) $$I{\left(t\right)}_{Decay}=I_{0}+A_{3}\left(e^{-t/\tau_{d1}}\right)+A_{4}\left(e^{-t/\tau_{d2}}\right)$$

The above equations present a bi-exponential function to scrutinize the nature of the rise and fall time where I_0_ is the dark current; A_1_, A_2_, A_3_, and A_4_ are constants; and τ_r1_, τ_r2_ and τ_d1_, τ_d2_ are RC time constants for the rise and decay of the photocurrent.

### 2.2. Pyro-Phototronic Effect in Metal–Semiconductor Junction

**Figure 2:**
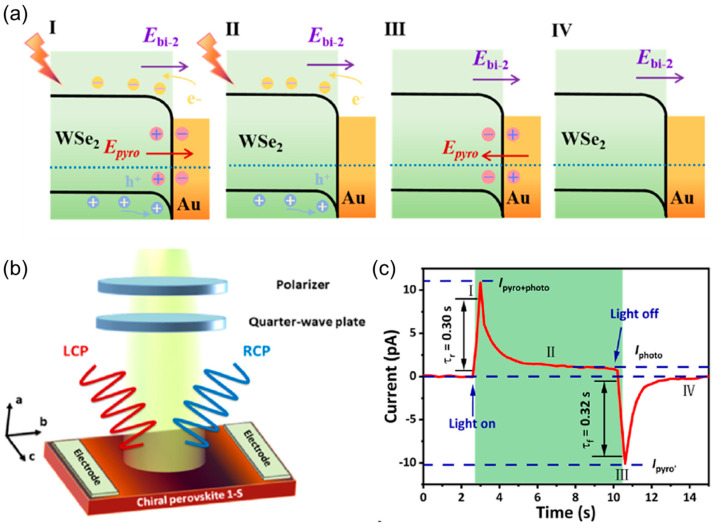
(**a**) Working mechanism of WSe_2_/Au Schottky junction [42], (**b**) device structure of chiral perovskite, and (**c**) pyro-phototronic I-t response of chiral perovskite [45].

Wang et al. presented self-powered PDs utilizing the Schottky interface between a Au and WSe_2_ layer. They demonstrated that the built-in electric field at the Au/WSe_2_ Schottky junction not only enhanced the asymmetry but also induced localized polarization through polar electric fields, thereby contributing to the pyroelectricity [42]. Figure 2a describes the four-stage I-t characteristic mechanism of the Au/WSe_2_ Schottky junction under 660 nm light. The instantaneous temperature increases within the Au/WSe_2_ Schottky junction under light exposure. This leads to a temperature difference that promotes polarized pyroelectric charge distribution along with the corresponding pyroelectric potential [43]. The alignment of the pyroelectric potential and built-in electric field effectively enhances the photogenerated carriers’ separation and facilitates their diffusion motion [44]. Similarly, when the light is turned off, the direction of pyroelectric potential becomes reversed, and an inverted peak of pyroelectric current appears in the output current.

Zhou et al. utilized chiral hybrid perovskites with the combined advantages of chiral materials and halide perovskites for designing circularly polarized light detectors [45]. For the circularly polarized light detectors, the polar materials possessing intrinsic spontaneous polarization generate photo-induced pyroelectric potentials under illumination [43,46]. This is mainly attributed to the temperature variations between the two terminals. Figure 2b shows the schematic diagram of the circularly polarized light detectors and the pyro-phototronic I-t response is shown in Figure 2c. The working mechanism of the transient photoresponses under a 520 nm laser has been discussed in detail. The change in electric polarization induces photo-excited pyroelectricity. During light exposure, the charge carrier separation of photogenerated carriers occurs via the built-in polarization field that stabilizes the I_photo_. The spontaneous polarization changes and the surface temperature increases via the light exposure that induces free charges on the surfaces and generates the positive I_pyro_. The PPE accelerates the process of generating photogenerated polarized charges and the devices regulate the charge transport at the interface.

**Figure 3:**
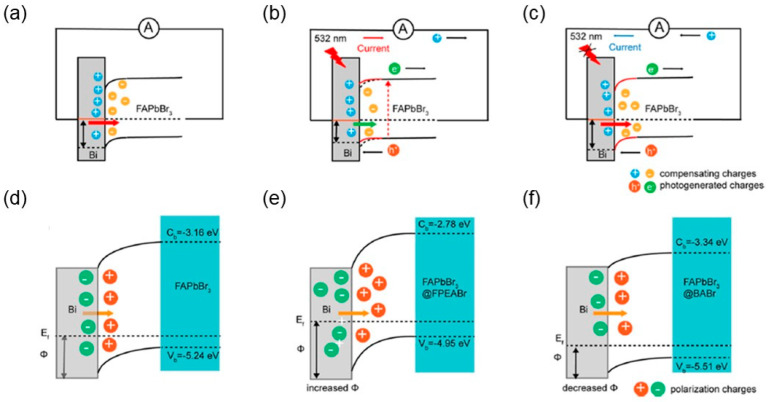
Mechanisms of the interfacial PPE modulated by surface passivation. (**a**) Schematic energy band diagrams of the FAPbBr_3_ MMB/Bi Schottky contact at (**a**) equilibrium, (**b**) light on, and (**c**) light off states. Band bending schematic and its resultant interfacial polarization at the Schottky junction interface for (**d**) FAPbBr_3_, (**e**) FAPbBr_3_@FPEABr, and (**f**) FAPbBr_3_@BABr MMB arrays [47].

Figure 3 demonstrates the working mechanism of enhancement in the interfacial PPE in the perovskite single crystal. Guo et al. utilized FAPbBr_3_, FAPbBr_3_ passivated with BABr, and FAPbBr_3_ passivated with 4-FPEABr [47]. These devices are denoted as FAPbBr_3_, FAPbBr_3_@BABr, and FAPbBr_3_@FPEABr, respectively, in Figure 3. These devices utilized the back-to-back contacted Schottky junction where the two back-to-back contacts do not exhibit similar asymmetric geometry of the device structures. Thus, the Schottky junction formation occurs at the p-type FAPbBr_3_ and Bi interface (Figure 3a,d). The depletion layer at the interface acts as the electric polarization layer with the positive and negative charges distributed on the FAPbBr_3_ and Bi sides, respectively. The Schottky junction acts as a barrier to prevent the device from suffering due to the recombination of electrons and holes. The interfacial PPE is also dominantly influenced by the barrier height and the built-in potential of the Schottky junction. The surface passivation of FAPbBr_3_ with BABr and 4-FPEABr (Figure 3b,e and Figure 3c,f) altered the band bending and the work function. Such modulation resulted in the increased space charge region at the interface exhibiting increased downward bending in FAPbBr_3_@FPEABr. However, FAPbBr_3_@BABr experienced a less effective barrier that redistributed the charge carriers through either tunneling or thermionic emission [48]. Such an occurrence of the effect severely impacted the pyroelectric effect of the FAPbBr_3_@BABr.

### 2.3. Pyro-Phototronic Effect in p-n Junction

**Figure 4:**
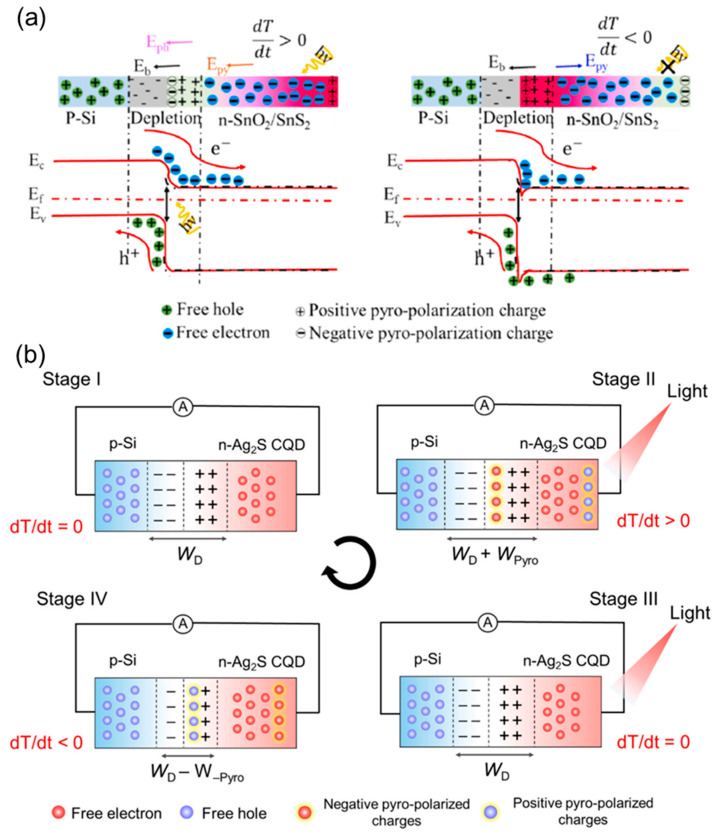
(**a**) Illustrative band diagrams of p-Si/SnO_2_/SnS_2_ PD in the presence of light in on and off states [57] and (**b**) schematic illustration of p-n junction CQD/Si PDs in four stages of photodynamic response [33].

Various semiconducting materials have been previously explored as pyro-phototronic PDs by utilizing multiple shapes/sizes of semiconducting materials. In PPE-based photodetectors, factors such as material size, shape, morphology, and density of polarizable charges are crucial for device performance. Smaller materials like nanoparticles or nanowires, with a larger surface-to-volume ratio, enhance sensitivity and enable faster response times due to reduced diffusion distances. The shape of these materials influences electric field distribution and charge separation efficiency. The morphology, crystalline structure, and surface roughness, impact dipole alignment. The high-quality crystals with fewer defects provide more consistent pyroelectric responses, while rough or disordered surfaces may cause performance issues. Finally, the density of polarizable charges determines the strength of the pyroelectric response, with higher charge densities improving sensitivity. However, excessive charge density may lead to polarization saturation, reducing effectiveness at higher temperatures or light intensities [26,35,49,50,51,52,53,54,55,56]. Very recently, Kumar et al. developed a self-powered 1D SnO_2_ nanoneedle/2D SnS_2_ nanoflower heterostructure PD due to elevated surface-to-volume ratios and unique geometrical features [57]. In the p-Si/SnO_2_/SnS_2_ heterostructure shown in Figure 4a, the occurrence of efficient charge separation at the p-n junction facilitated high photocurrent due to the band structure favoring the electron–hole pair generation and collection. The utilization of n-Si/SnO_2_/SnS_2_ heterostructure faces several challenges including the existence of recombination centers at the interface. These recombination centers hinder the charge separation process. Ultimately, photocurrent is reduced in the n-Si/SnO_2_/SnS_2_ heterostructure. The n-n heterostructure does not promote pyroelectric potential generation due to a lack of inherent asymmetry which limits charge distribution and electric fields. Moreover, the built-in electric field remains absent, and symmetric carrier concentration limits the pyrocurrent contribution to the PD’s performance. Bhatt et al. developed p-n junction PPE-induced PDs using solution-processed CQDs [33]. The use of Ag_2_S CQDs in the CQD/silicon PDs, influenced by the PPE, demonstrated excellent photodetection in the NIR. When the bulk centrosymmetric crystal structure is confined to 0D, 1D, or 2D, structural distortion takes place.

The confinement of centrosymmetric crystal structure inherently lacks inversion symmetry, resulting in spontaneous polarization change [33,56,58,59,60,61]. Figure 4b illustrates the operational mechanism of the CQD/silicon PD working mechanism under the influence of the PPE. The photoelectric effect and PPE are evidenced by variations in depletion width. The depletion width increases or decreases upon the instantaneous exposure to or removal of light from the device. The depletion width varies relative to the equilibrium state of the p-n junction upon sudden exposure to or removal of light. This variation induces positive and negative spikes in stages II and IV, respectively, as depicted in Figure 4b.

## 3. Photodetectors Based on Pyro-Phototronic Effect

### 3.1. Broadband Photodetectors

**Figure 5:**
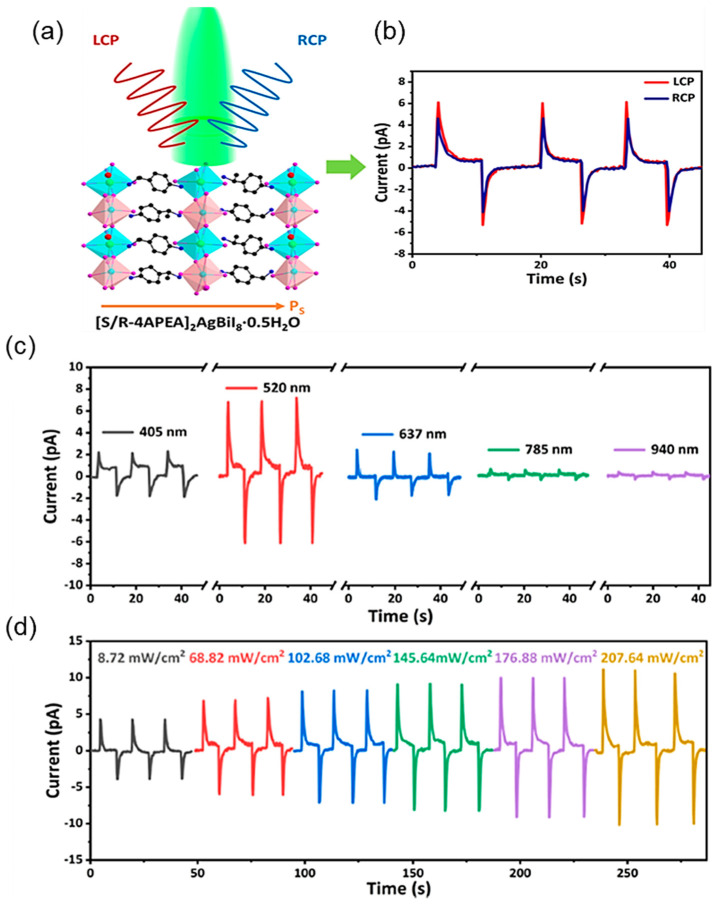
(**a**) Layered structure of S/R-[(4-aminophenyl)ethylamine]_2_AgBiI_8_·0.5H_2_O. (**b**) LCP/RCP pyro-phototronic responses under 520 nm laser with 68.82 mW/cm^2^ at zero bias. (**c**) Wavelength-dependent response of 1-S and (**d**) power-density-dependent photoresponses of 1-S at 520 nm [45].

Ma et al. developed Ag_2_Se/Si heterojunction PDs based on photovoltaic and pyroelectric coupled effects. They demonstrated self-powered broadband photodetection ranging from 405 to 1064 nm [62]. The reversible phase transition properties between the low-temperature β-phase and high-temperature α-phase result in the alteration of the material’s physical and chemical properties. These properties could be helpful for the generation of PPE in the Ag_2_Se crystal. Studies on the thickness dependence of Ag_2_Se thin films revealed reduced electron transport distance from the Ag_2_Se surface to the Si substrate. Such reduced distance can decrease the recombination rate and enhance the laser position-dependent lateral photovoltage response. Among the Ag_2_Se thin films with thicknesses ranging from 10 to 70 nm, the 20 nm thickness exhibited the highest lateral photovoltage response. The faster response speed is attributed to the four-stage dynamic behavior under the PPE. Wang et al. utilized a graded p-Si/n-Zn_1−x_Mn_x_O heterojunction for UV–vis–NIR detection wherein the Mn doping grading induced a stronger polarization field, improving the PPE for 300–1100 nm detection and the pyroelectric effect for 1100–1700 nm detection [63]. The graded strain-generated polarization field induced a band tilt in the n-Zn_1−x_Mn_x_O, resulting in a slight increase in hole concentration on the Si substrate, thereby generating a local field. The larger built-in electric field of the graded p-Si/n-Zn_1−x_Mn_x_O heterojunction induces a significant symmetric broken and large pyroelectric polarization field. Consequently, the graded strain can effectively enhance the pyroelectric effect by enlarging the built-in field. The 2D lead-free chiral–polar double perovskites, 1-S/1-R-based PPE-induced PDs were developed by utilizing chiral organic ligands into metal halide frameworks (Figure 5a) [45]. The 1-S possesses the coupling effect of chiroptical phenomena and PPE which is very useful for detecting circularly polarized light. Figure 5b demonstrates the photocurrent under 405 nm of left and right circularly polarized (LCP and RCP light) indicating outstanding resolution between LCP and RCP light. Additionally, PDs exhibit a broadband pyro-phototronic response from ultraviolet (UV) to infrared (IR) under a self-biased condition with 68 mW/cm^2^ power density. The light-induced pyrocurrent demonstrated a stronger dependency of PDs on the incident wavelength under fixed power intensity and showed a smaller change in electric polarization induced by longer wavelength incidence. The devices exhibited a light-intensity-dependent I-t curve under a 520 nm laser at zero bias (Figure 5c,d). The increasing incident power enhanced the photocurrent and pyrocurrent in the devices and exhibited obvious power-intensity-dependent behavior.

**Figure 6:**
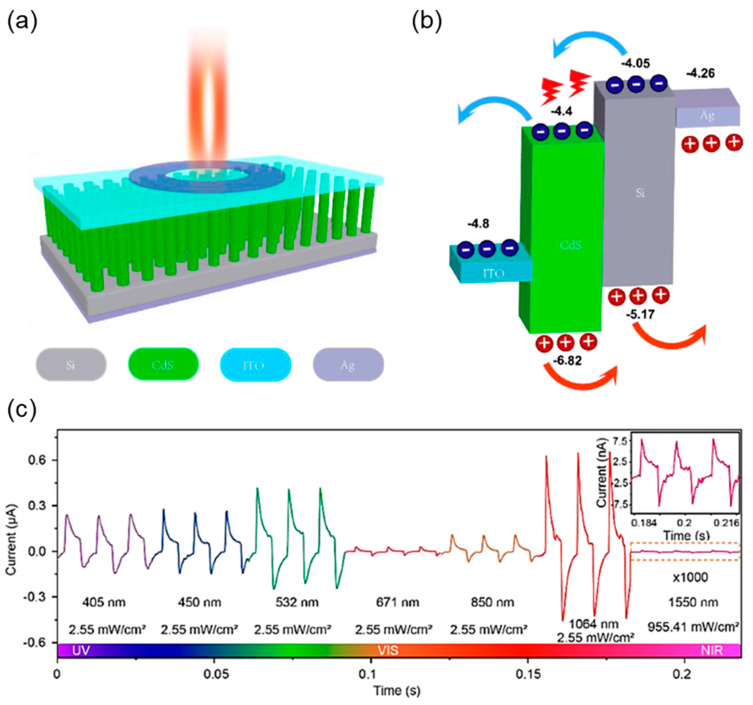
(**a**) Schematic diagram of n-CdS nanorod/p-Si heterojunction PD devices. (**b**) Energy band diagram of n-CdS nanorod/p-Si heterojunction wherein energies are in electron volts, with the electron energy in vacuum as a reference. (**c**) Transient I-t curves of n-CdS nanorod/p-Si heterojunction PD under different wavelengths [68].

Li et al. explored one of the third-generation pyroelectric semiconductor materials: CdS, which possesses a non-centrosymmetric wurtzite crystal structure [64]. This work demonstrated excellent PPE performance ranging from 365 to 1310 nm in p-Si/n-CdS heterojunctions under self-biased conditions. The devices also exhibited ultrafast response speed over a broadband response under self-biased conditions, indicating overall excellent photodetection performance due to the PPE. Earlier, various research groups explored CdS with a non-centrosymmetric wurtzite crystal structure for PPE-induced photodetection. They demonstrated various types of modifications in terms of synthesis and surface modification and by forming heterostructures with other promising materials [65,66,67]. Ren et al. developed broadband CdS nanorods/planar-Si PDs by synthesizing CdS nanorods using a simple, low-cost hydrothermal synthesis route [68]. Figure 6a,b show the schematic diagram of the n-CdS nanorod/p-Si heterojunction and its respective band alignments. The CdS nanorods were grown along with the c-axis orientation on planar Si, which induced a pyroelectric field under temperature variation. It significantly enhanced the carrier transport and separation in the heterojunction. Figure 6c shows the I-t response of the device observed for various laser wavelengths ranging from 405 to 1550 nm. The devices exhibit photovoltaic and PPEs for laser wavelengths below 1550 nm, whereas the photocurrent was absent when the I-t response was measured at a 1550 nm wavelength due to the absence of photogenerated charge carriers. Moreover, the temperature-dependent I-t curves measured at 1064 nm ranging from 25 to 150 °C also demonstrated a decrease in I_photo_ + I_pyro_ and I_photo_ + I_pyro_ − I_pyro’_ with increasing ambient temperature.

Recently, Nataraj et al. investigated the role of selectively patterned black silicon on light absorption and the occurrence of PPE [69]. Various types of textured surfaces were created. This includes microwells, hexagons, grating, broad pillars, inverted hemispheres, toroids, inverted toroids, stars, and flat-top hemispheres of black silicon. In comparing the electrical characteristics among planar, black silicon, and microwell-patterned devices, microwell-patterned devices exhibited PPEs under a self-biased condition.

**Figure 7:**
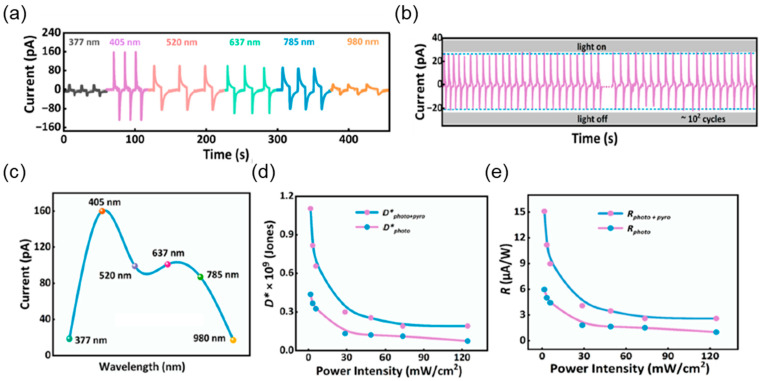
(**a**) I-t curves of the (1,3-BMACH)BiBr_5_ devices under light illumination of different wavelengths. (**b**) Stability test of photovoltaic and pyroelectric current under 405 nm. (**c**) Wavelength-dependent current and light-intensity-dependent (**d**) D* and (**e**) R of the device based on (1,3-BMACH)BiBr5 single crystal [70].

Fu et al. adopted the multi-effect coupling strategy for enhancing the self-powered photoresponse in polar, lead-free hybrid perovskites (1,3-BMACH)BiBr_5_ [1,3-BMACH = 1,3-bis(aminomethyl)cyclohexane] [70]. Figure 7a shows the I-t response of the single-crystal devices based on (1,3-BMACH)BiBr_5_ for wavelengths ranging from 377 nm to 980 nm, respectively. The I-t response at 377 nm consisted only of pyrocurrent due to limited light absorption whereas the I-t response at 405 nm was quite pronounced, displaying stability even after 100 cycles of operation as shown in Figure 7b. Based on the wavelength-dependent I-t response measurements, the device exhibited a maximum current generated at 405 nm as described in Figure 7c. Due to the occurrence of photovoltaic and pyroelectric effects, (1,3-BMACH)BiBr_5_ can achieve a photoresponse beyond its light absorption range. The power-intensity-dependent I-t response demonstrated a more significant ΔT occurrence with increasing power intensity. R and D* were both enhanced by ∼260% after coupling with PPE based on Figure 7d,e.

Yu et al. developed Si/ZnO heterojunction PDs by utilizing magnetron sputtering to deposit c-axis-oriented ZnO films for broadband photodetection [71]. The excellent broadband photodetection of the Si/ZnO heterojunction PDs is attributed to quite uniform and dense ZnO film. The ZnO film deposition by sputtering reduced the gas molecules’ adsorption in the films. The improved PPE might be attributed to the suppressed in-plane thermal diffusion and the increased transient temperature gradient in the out-of-plane orientation. Moreover, the suppressed dark current by the Si/ZnO barrier near the interface improves the detectivity of the devices even at low power intensities.

### 3.2. Narrowband Photodetectors

#### 3.2.1. Ultraviolet Photodetectors

As discussed in the introduction, various ZnO-based PDs have been previously explored. However, the research still lacks several aspects of ZnO that could shed light on improving the pyro-photototronic effect at the advanced level. Qiao et al. explored the role of the ZnO NW’s length dependency on inducing the PPE in ZnO/Si heterojunction PDs [55]. The variation in the ZnO NW’s length from 200 to 1000 nm played a fundamental role in light absorption and promoting the transport/separation of photogenerated carriers. It also showed its vital impact on how the distribution of polarization charges/the strength of the polarization field occurs under transient illumination [72,73,74]. The results demonstrated that the ZnO NWs with a 200 nm diameter experienced maximum current responses and ultimately resulted in the best responsivity and detectivity. However, the maximum responsivity ratio was obtained for a 500 nm NW length. Thus, the output decreased for NW lengths below or above 500 nm due to low light absorption and reduced charge separation efficiency, respectively. Rabha et al. reported the PPE in Al/nanostructured porous silicon multi-layers by varying the layers of porous silicon from 13 to 25. They ultimately varied the thicknesses from 6.5 to 11.5 µm [75]. Tang et al. presented an organic–inorganic heterojunction PD by utilizing bismuth oxychloride (BiOCl) and TiO_2_ nanorods for UV light detection [76]. BiOCl/TiO_2_ exhibited better performance due to a built-in electric field created between BiOCl and TiO_2_. The utilization of BiOCl promoted efficient charge separation in TiO_2_ NRs and suppressed electron–hole pair recombination.

Tang et al. adopted a novel approach by utilizing TiO_2_ nanoarrays and the conducting polymer poly(3,4-ethylenedioxy selenophene) (PEDOS) for inducing PPE in UV PDs [77]. The HCl- and H_2_SO_4_-treated TiO_2_ nanorods formed p-n junctions with PEDOS wherein the HCl and H_2_SO_4_ treatment dramatically influenced the p-n heterojunction properties. The HCl treatment improved the carrier concentration by introducing surface oxygen vacancies on the TiO_2_ NR’s surface. On the other hand, the H_2_SO_4_ treatment improves the interaction in the p-n heterojunction. The H_2_SO_4_ treatment also increased the surface area of TiO_2_ nanorods, thereby providing more convenient charge transport along with suppressed recombination and resulting in higher conductivity [23,78,79,80]. Jiang et al. utilized solvent-free 2D SnSe films exhibiting exceptional light–matter interaction and versatile electronic characteristics [81]. The SnSe films were used with the metal–semiconductor–metal configuration on a paper substrate and demonstrated good responsivity at a 254 nm wavelength. Zhou et al. developed self-powered UV PDs by utilizing a 2D Dion–Jacobson (DJ) phase lead-free double perovskite known as the HAAg_0.5_Bi_0.5_Br_4_ (HA^2+^ = histammonium) perovskite [82]. Bi^3+^ and Ag^+^ were used to construct the 2D DJ perovskite with organic Histamine (HA) and device structure: ITO/PEDOT:PSS/HAAg_0.5_Bi_0.5_Br_4_/PC_61_BM/BCP/Ag was fabricated for photodetection. The pyroelectric effect in some of the 2D layered perovskite materials is mainly attributed to asymmetry that helps in producing electrical polarization under temperature fluctuations.

#### 3.2.2. Visible Photodetectors

**Figure 8:**
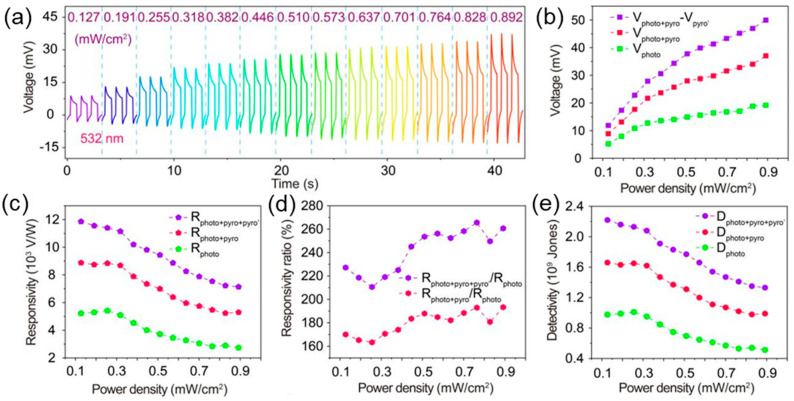
(**a**) Transient V-t curves of the 50-nm -thick ZnO PD under a light-intensity-dependent wavelength of 532 nm. The extracted power-density-dependent (**b**) photovoltage, (**c**) responsivities, (**d**) responsivity ratios, and (**e**) detectivity [32].

Lim et al. developed a single-phase perovskite (C_4_H_9_NH_3_)_2_PbI_2_Br_2_-based planar PD facilitated by light-induced phase segregation [83]. The PDs exhibited the occurrence of light-induced PPE ranging from 405 to 635 nm. The formation of I- and Br-rich domains in (C_4_H_9_NH_3_)_2_PbI_2_Br_2_ due to phase segregation increases the polarity, which in turn induces a PPE. Figure 8 displays the photodetection characteristics of an ITO/ZnO/CdS/MAPbI_3_/Spiro-OMeTAD heterojunction PD by varying ZnO thickness from 0 to 95 nm at the CdS/ITO interface [32]. Figure 8a shows the light-intensity-dependent transient response curves under a 532 nm laser. The extracted photovoltage values demonstrated the gradual increase in photovoltage with increasing power density as shown in Figure 8b. The power-density-dependent responsivity, responsivity ratio, and detectivity are shown in Figure 8c–e. From a single cycle of the transient response, the rise/fall time was measured to be 28/29 ms. The wavelength-dependent responsivity and detectivity validated that the PDs exhibited excellent performance in the visible range.

#### 3.2.3. Infrared Photodetectors

**Figure 9:**
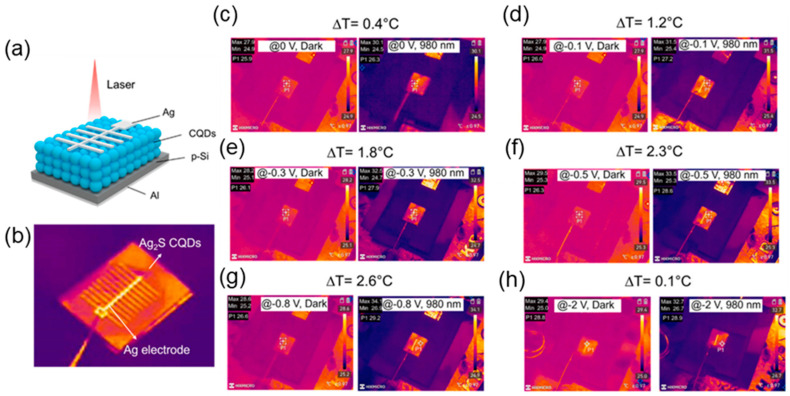
(**a**) Schematic diagram of Ag_2_S CQD/p-Si PDs: (**b**) thermal camera images of CQD/Si PDs and thermal images in the dark and under 980 nm light irradiation at (**c**) 0 V, (**d**) −0.1 V, (**e**) −0.3 V, (**f**) −0.5 V, (**g**) −0.8 V, and (**h**) −2 V [33].

In 2023, Ma et al. developed Ag_2_Se/Si heterojunction PDs by utilizing an Ag_2_Se thin film for tuning the PPE. Although the devices showed a broadband response, they exhibited excellent photodetection at 1064 nm among the broad range of wavelengths. Such an excellent response to 1064 nm was examined under the frequency of light and light intensity, demonstrating the ability of a 70 nm Ag_2_Se thin film to induce PPE [50]. Fathi et al. demonstrated an approach to designing heterojunction-based PDs for detecting a 950 nm wavelength by utilizing p-type CuInS_2_/p-type Cu_2_SnS_3_ nanocrystal layers. The CuInS_2_ nanocrystals provided excellent light absorption up to 1000 nm and Cu_2_SnS_3_ nanocrystals provided excellent light absorption beyond 800 nm and up to 2000 nm [84]. The overlapping light absorption of both nanocrystals in the 800–1000 nm range played a vital role in excellent charge carrier generation under a 950 nm light source. Although CuInS_2_ and Cu_2_SnS_3_ nanocrystals are centrosymmetric materials, the heterojunction PDs benefit from the polar symmetry induced by size confinement. Figure 9 demonstrates the thermal imaging recorded for CQD/Si PDs under the influence of applied bias voltage. Figure 9a,b show the device schematic and the top view of the thermal imaging of the device structure. The thermal imaging analysis from Figure 9c–h describes the temperature variation upon exposure to 980 nm light on the device. The ΔT between dark and 980 nm irradiation significantly increased as the bias increased from 0 to −0.8 V, demonstrating maximum ΔT occurring at −0.8 V. The ΔT became minimal beyond −0.8 V, indicating the occurrence of PPE under the influence of the applied bias.

Table 1 summarizes research on PPE, covering materials, device structure, operating wavelength, and device performance parameters such as responsivity, detectivity, and rise/fall time. This comparative approach helps researchers comprehensively evaluate the progress of pyro-photototronic effect research and formulate strategic plans for future studies. The literature table demonstrates photodetection performance parameters for various types of device structures. Most PPE-induced PDs operate within the UV–visible wavelength range, and there is a notable lack of utilization of PPE in infrared (IR) PDs. The rise and fall times for PPE-induced photodetection in the near-infrared (NIR) range were significantly faster (up to microseconds) when utilizing Ag_2_Se and CdS as semiconducting materials contributing to the PPE. The quantitative comparison of photodetection performance with and without PPE also provides key insights into improving the performance parameters. For example, Li et al. reported that both; photoresponsivity and specific detectivity are enhanced by 23.3 times compared to those based solely on the photovoltaic effect [64]. Zhang et al. observed the improvement in dual-polarity photocurrents in the CdS layer by calculating maximum enhancement factors of 120%, 343%, 1167%, 1577%, and 1896% at 405, 450, 532, 650, and 808 nm, respectively [85]. Bhatt et al. reported the enhancement in the detectivity of the CQD/silicon PDs by a factor of 17 at 980 nm [33]. Similarly, Bhatt et al. also observed that Si/MoO_3−x_ 2D-sheet heterojunctions significantly enhanced the responsivity and detectivity up to 86% via the PPE [56]. Conversely, most PDs operating in the UV–visible range still exhibit rise and fall times in the millisecond range, which necessitates further improvement through the application of PPE. Similarly, UV PDs need to achieve higher detectivity, well beyond 10^12^ Jones, to facilitate their integration with the latest technology. Pyro-phototronic-based PDs offer high responsivity, self-powered operation, and broadband sensitivity but face challenges in stability, efficiency, and scalability. The PPE is mainly observed in non-centrosymmetric materials like ZnO, CdS, GaN, and perovskites, limiting material choices and advancements [86]. Some centrosymmetric materials, such as Ag_2_S CQD, MoO_3_, and SnS, exhibit this effect due to structural distortion when reduced to 0D, 1D, or 2D. These may lack inversion symmetry and allow spontaneous polarization changes associated with pyroelectricity [33,56,57].

## 4. Challenges and Future Scope

In this review, we discuss a detailed approach to improving self-powered photodetector efficiency by utilizing polarization charges in various materials and heterojunction devices. This review examines the fundamental mechanism of PPE and discusses recent advances in comprehensive research on various nanostructured materials. PPE in various materials and heterojunction devices has been extensively explored in optoelectronics and energy applications. The study of optoelectronic devices based on PPEs undoubtedly needs to be expanded in numerous directions. Advancements in PDs are crucial for military, medical, remote sensing, and communication applications. Heterojunction engineering enhances photodetection by minimizing carrier recombination and optimizing responsivity, quantum efficiency, and photogain. The discovery of new materials and innovations in material properties will significantly enhance PD performance, elevating it to unprecedented levels.

(1) The performance of devices based on PPE is greatly influenced by the material’s size, shape, and morphology. To improve device performance, it is also necessary to systematically demonstrate the effect of size, shape, and morphology on the density of polarizable charges, which is currently understudied. The reduced 3D structure in 0D, 1D, and 2D materials exhibits the PPE due to the lack of inversion symmetry, which allows the emergence of spontaneous polarization changes associated with pyroelectricity. However, the discontinuity in their 3D structure limits carrier transport, resulting in lower overall PD performance compared to 3D materials. Therefore, it is necessary to optimize the performance of low-dimensional materials through other methods, such as device structure design. A major drawback is the low pyroelectric coefficients in many semiconductors, hindering strong electrical signal generation in response to ΔT. Stability issues arise as perovskites degrade with moisture, oxygen, and heat, while ZnO and GaN degrade under prolonged UV exposure. Controlling the pyro-phototronic response is challenging due to its dependence on unpredictable temperature variations, making real-world applications inconsistent. ZnO’s low charge carrier mobility limits performance compared to high-speed semiconductors like Si or GaAs. Fabrication requires precise control over defects, doping, and crystal structure, increasing complexity and cost. Scalability is difficult due to material uniformity and processing constraints. Energy loss and efficiency trade-offs occur due to interactions between pyroelectricity, photoconductivity, and the photovoltaic effect, complicating optimization.
(2) Only a few semiconductor materials have been discovered to date for this application. More and more novel materials should be developed and investigated to improve PPE in optoelectronic devices. Certain material properties are crucial for the performance of PPE-based PDs. High pyroelectric coefficients in materials like ZnO and GaN enable strong charge generation through temperature-induced polarization, enhancing signal strength and sensitivity. Wide-bandgap semiconductors such as ZnO and GaN provide excellent thermal and electrical stability, reducing dark current and improving the signal-to-noise ratio (SNR). The synergistic coupling of pyroelectric and photoelectric effects in wurtzite-structured semiconductors facilitates efficient charge separation and amplification. These properties enhance photodetection without external biasing. High-thermal-conductivity materials like GaN dissipate heat efficiently, preventing localized overheating and ensuring stable operation. The complex interplay of pyroelectric, photoconductive, and photovoltaic effects can lead to energy losses if not optimized. And, it would require external modulation to maintain efficiency and complicate system design.
(3) The pyro-phototronic effect is successfully demonstrated in heterojunction devices; it is worth noting that energy band alignment plays an important role in device performance improvement. A perfect energy band alignment can result in extremely high utilization efficiency of polarization charges at the interface. Perovskites offer flexibility, making them ideal for wearable and stretchable PDs, expanding applications in biomedical sensing and next-generation optoelectronics. Additionally, scaling and fabricating wide-bandgap materials presents compatibility challenges. It is complicated when considering their compatibility with CMOS technology commonly used in AI chips and IoT sensors. Stability is another concern, as materials like perovskites degrade under moisture and heat, limiting their suitability for long-term outdoor use. The complex interplay of pyroelectric, photoconductive, and photovoltaic effects can lead to energy losses if not optimized. Hence, it requires external modulation to maintain efficiency, complicating the system’s design.
(4) No theoretical simulation study of the PPE in heterojunction devices is available in the literature. A lot of attention is required in this field to uncover many hidden aspects related to energy band alignment. Hence, simulation studies will help in developing suitable novel materials. Here, we have discussed the applications of these PDs in optical communication, automotive engineering, military, image sensing, thermal imaging, night vision, biochemical analysis, motion detection, space exploration, etc. It is necessary to develop and demonstrate more inventive and diverse potential applications. Additionally, multiple couplings of PPE with ferroelectricity and piezoelectricity improve the performance of self-powered PDs. However, the multi-effect coupling mechanism with PPE remains unclear. More detailed mechanisms, such as carrier transport mechanisms and electronic properties, need to be investigated.
(5) The integration of pyro-phototronic PDs into AI and IoT systems can offer significant potential due to their enhanced sensitivity, low power consumption, and multi-functional capabilities. Despite the promising integration of pyro-phototronic PDs into AI and IoT systems, several compatibility concerns must be addressed. The complex signals generated by these detectors are influenced by both: thermal and optical stimuli. They require specialized AI algorithms for accurate interpretation, while existing AI and IoT devices are built around standardized PD interfaces, necessitating custom signal processing. Future research on pyro-phototronic PDs is set to boost their stability, performance, and range of applications. Key innovations include creating hybrid pyroelectric–photonic materials, doping ZnO or GaN to improve charge carrier mobility, and developing flexible, wearable sensors. Advanced device designs will focus on neuromorphic PDs for AI, multi-wavelength detection, and hybrid energy systems that combine PPEs with other energy-conversion technologies. Integrating AI and machine learning will enhance signal processing, enable real-time sensing, and create adaptive, self-calibrating sensors for dynamic environments. Efforts to scale and integrate with CMOS technology include developing silicon-compatible pyro-phototronic sensors, large-area sensor arrays, and advanced nanofabrication techniques. Emerging applications span biomedical imaging, space exploration, quantum optoelectronics, and defense, with potential uses in IR biometric sensing, low-power radiation detection, THz communication, and smart camouflage for security. These advancements open up exciting new possibilities for pyro-phototronic technologies across various fields.

## Figures and Tables

**Table 1:** Comparison of performances of pyro-phototronic-effect-based PDs under self-biased conditions.

Device Structure	Spectral Range [nm]	Operating Wavelength [nm]	Light Intensity [mW/cm^2^]	D* [Jones]	R (mA/W)	tr/tf [s]	Ref.
Ag_2_Se/Si	405–1064	1064	20	-	-	3 µ/5 µ	[62]
p-Si/n-Zn_1−x_Mn_x_O	300–1700	900	-	4 × 10^13^	140	3.4 m/4.1 m	[63]
p-Si/n-CdS	365–1310	980	1.7	-	23.3 × 10^−3^	70 µ/90 µ	[64]
ZnO/PEDOT:PSS	360–1550	360	-	8.17 × 10^10^	0.19	-	[55]
ZnO/Si	360–1550	360	-	2.44 × 10^11^	0.56	-	
Al: PS-ML: p^+^-Si	-	365	-	-	97147	0.4/-	[75]
Ag_2_Se/Si	405–1064	1064	0.64	17.5 × 10^10^	43.32	64 µ/62 µ	[50]
CIS/CTS	-	950	-	6.5 × 10^11^	42.5	49.6 m/34.7 m	[84]
PEDOS/TiO_2_ (HCL)	-	365	-	3.6 × 10^11^	58.7	0.08/0.079	[77]
n-CdS nanorods/p-Si	405–1550	1064	2.55	1.31 × 10^10^	64.8	0.19 m/0.29 m	[68]
Chiral–polar double perovskites	-	520	68	0.5 × 10^3^	0.01 × 10^−6^	0.3/0.32	[45]
p-black silicon/ZnO/SiNP	-	Solar simulator	100	3.5 × 10^9^	-	70 m/110 m	[69]
Au/WSe_2_/Ta_2_NiS_5_/Au	405–1064	660	0.01	2 × 10^12^	121	17 m/33 m	[42]
Polar hybrid perovskite	X-ray-NIR	405	-	1.1 × 10^9^	15.1 × 10^−3^	75 µ/336 µ	[70]
(BA)_2_PbI_2_Br_2_	405–635	405	-	1.49 × 10^12^	0.12 × 10^3^	654 m/296 m	[83]
FAPbBr_3_@FPEABr	-	532	0.13 × 10^−3^	1 × 10^10^	300	-	[47]
SnO_2_/SnS_2_	365–850	365	0.008	1.32 × 10^8^	3.65	37 m/40 m	[57]
Si/ZnO	355–1550	405	500 × 10^−6^	7.7 × 10^12^	550.6	0.13 m/0.12 m	[71]
TiO_2_ NRs/BiOCl/ PEDOS	-	365	0.32	39.3 × 10^10^	108.8	0.080/0.079	[76]
MAPbI_3_	360–1550	532	0.892	2.22 × 10^9^	1.19 × 10^4^ V/W	28 m/29 m	[32]
Ag/β-Ga_2_O_3_	200–980	450	4	568.6 cm Hz^1/2^/W^−1^	20.2 × 10^−9^	-	[39]
TO/HAAg_0.5_Bi_0.5_Br_4_	265–420	365	8.96	6 × 10^12^	0.9	1.32 m/0.11 m	[82]

## Data Availability

No new data were created or analyzed in this study.

## Abbreviations

The following abbreviations are used in this manuscript:

PDs	Photodetectors
AI	Artificial intelligence
IoT	Internet of Things
CQDs	Colloidal quantum dots
1D	One-dimensional
2D	Two-dimensional
3D	Three-dimensional
UV	Ultraviolet
NWs	Nanowires
PPE	Pyro-phototronic effect
CPL	Circularly polarized luminescence
(S-BPEA)_2_FAPb_2_I_7_	(S)-1-4-Bromophenylethylammonium
FA	Formamidinium
LDR	Linear dynamic range
EQE	External quantum efficiency
D*	Detectivity
R_λ_	Responsivity
S	Sensitivity
t_r_/t_f_	Rise/fall time
LCP	Left circularly polarized
RCP	Right circularly polarized
(1,3-BMACH)BiBr_5_	1,3-bis(aminomethyl)cyclohexane
BiOCl	Bismuth oxychloride
PEDOS	Poly(3,4-ethylenedioxy selenophene)
DJ	Dion–Jacobson
HA	Histamine
ΔT	Temperature change
IR	Infrared
NIR	Near-infrared
SNR	Signal-to-noise ratio
